# Efficacy of decellularized extracellular matrix (dECM) for articular cartilage repair in osteoarthritis (OA): a systematic review and meta-analysis

**DOI:** 10.1186/s13018-025-05881-2

**Published:** 2025-05-16

**Authors:** Tao Wang, Mingyang Jiang, Sichang Wu, Ke Zhang, Raul Romero Del Rey, Ruqiong Wei, Raquel Alarcón Rodríguez

**Affiliations:** 1 Faculty of Health Sciences, University of Almeria, Carretera Sacramento s/n, La Canada, Almería, 04120 Spain; 2 Department of Bone and Joint Surgery, The First Affiliated Hospital of Guangxi Medical University, Nanning, China; 3 The Second Clinical Medical College of Guangxi Medical University, Nanning, China; 4 Department of Rehabilitation Medicine, The First Affiliated Hospital of Guangxi Medical University, Nanning, China

**Keywords:** Osteoarthritis, Decellularized extracellular matrix, Cartilage repair, Systematic review

## Abstract

**Background:**

Osteoarthritis (OA) is a common degenerative joint disease causing chronic pain, disability, and mobility limitations, severely affecting quality of life. Traditional treatments like physical therapy and surgery often have limited efficacy due to side effects, incomplete recovery, and disease progression, highlighting the need for innovative therapies.

**Methods:**

We searched PubMed and Embase from January 1, 2010 to November 1, 2024, preliminary included studies involving animal experiments on the therapeutic effects of decellularized extracellular matrix (dECM) and its derived materials on cartilage defect. After removing duplicates, we conducted a bibliometric analysis. Following the exclusion and evaluation of literature, the random/fixed effects model was employed to perform meta-analysis and obtain Weighted Mean Difference (WMD) of Osteoarthritis Research Society International (OARSI) score and International Cartilage Repair Society (ICRS) score between the dECM treatment group and corresponding control group. We verify the robustness of the results through subgroup analysis and sensitivity analysis, with heterogeneity assessed by Q-test and quantified via *I*^2^ values.

**Results:**

We included a total of 10 studies, of which 7 were used for ICRS-based meta-analysis and 3 were used for OARSI-based meta-analysis. The combined mean ICRS of dECM treatment group/control group resulted in an WMD of 2.45 (95% CI: 1.07 to 3.84; *I*^2^ = 97.4%); *P*-value < 0.001). Meanwhile, the combined mean OARSI of dECM treatment group/control group resulted in an WMD of -1.65 (95% CI: -3.63 to 0.34; *I*^2^ = 97.3%). The subsequent funnel plot confirmed the low publication bias of the above results.

**Conclusions:**

Based on the dual-index meta-analysis, the dECM and relative derivatives have been proved to possess significant cartilage repair function in OA, which can be further explored in tissue regeneration filed.

**Supplementary Information:**

The online version contains supplementary material available at 10.1186/s13018-025-05881-2.

## Background

Osteoarthritis (OA) is a common degenerative joint disease characterized by chronic pain, disability and impaired mobility, with an incidence of up to 16.05%, affecting more than 500 million people worldwide [1]. Therapeutic strategies for OA can be broadly categorized into two main approaches: non-surgical and surgical interventions. The non-surgical management includes conventional methods such as physical therapy and anti-inflammatory medications, as well as biological therapies and other emerging treatment modalities [2–12], while surgical options primarily involve procedures like joint replacement. However, both approaches present significant limitations — non-surgical management is often hampered by transient therapeutic efficacy and symptom recurrence, whereas surgical interventions carry inherent risks of trauma and require prolonged recovery periods. However, both approaches exhibit significant limitations—non-surgical management often suffers from transient efficacy and symptom recurrence, while surgical options carry inherent risks of trauma and prolonged recovery periods. Notably, regardless of the chosen treatment modality, common challenges include suboptimal therapeutic outcomes, adverse effects, and persistent disease progression, highlighting the critical need for developing novel treatment strategies [1].

The decellularized extracellular matrix (dECM) is a natural biomaterial that retains the original three-dimensional structure and chemical composition of tissues after the removal of cellular components. It exhibits high biocompatibility and bioactivity, enabling it to mimic the natural tissue microenvironment and provide ideal conditions for cell adhesion, proliferation, migration, and differentiation [13]. Moreover, dECM retains natural proteins, glycosaminoglycans, and other key biological factors, offering crucial support for tissue repair and functional reconstruction [14]. The greatest advantage of dECM lies in its multifunctionality, with wide applications in tissue engineering and regenerative medicine. For instance, in cardiac tissue engineering, heart-derived dECM aids in reconstructing myocardial tissue with functional contractile capacity [15]. In liver models, dECM effectively supports the maintenance and maturation of hepatocyte functions through its rich biological signals [16]. Due to its natural properties and functional versatility, dECM demonstrates vast application prospects in regenerative medicine, drug development, and tissue engineering.

Recent advances in regenerative medicine have highlighted the potential of dECM as a novel therapeutic agent for OA. Previous studies have suggested that dECM possesses regenerative properties that promote cartilage repair and reduce inflammation in OA, thus positioning it as a promising candidate for innovative treatment solutions [17, 18]. Recent studies have explored the underlying therapeutic mechanisms of dECM in OA. For instance, Annamalai et al. dECM provides a favorable tissue-like microenvironment for the growth of mesenchymal stem cells, thereby promoting the differentiation of chondrocytes [19]. Additionally, the dECM hydrogel is a good scaffold to carry various nanoparticles and components with therapeutic efficacy against OA [20]. More importantly, dECM hydrogel can exhibit sustained-release capabilities, prolonging the residence time of these cargos within the joint cavity [21]. Therefore, dECM hydrogel with high biocompatibility and modifiability can be combined with various therapeutic agents and strategies, exhibiting greater versatility and development potential.

Despite the growing interest in dECM, a significant gap remains in the comprehensive understanding of its efficacy and mechanisms of action in OA. Although several studies have indicated that dECM may serve as an effective approach for repairing articular cartilage in OA, a comprehensive statistical analysis of its efficacy is still required to provide robust evidence for clinical translation. Conducting systematic literature reviews and meta-analyses can provide clearer insights into the clinical benefits of dECM, as well as identify potential areas for further research [22, 23].

The methodologies employed in this study include systematic literature review and meta-analysis, which facilitate a robust assessment of dECM’s therapeutic effects in preclinical models. Based on standardized scoring systems, included Osteoarthritis Research Society International (OARSI) score and International Cartilage Repair Society (ICRS) score, this research aims to evaluate the comparative outcomes of dECM treatment against control groups, thereby elucidating its potential as a viable alternative to conventional therapies [24, 25]. The insights gained from this research may contribute to the development of novel therapeutic strategies aimed at improving patient outcomes in OA and management of related conditions [26, 27].

## Methods

### Literature search

Two researchers independently conducted a systematic literature search in two major academic databases: the PubMed, and Embase databases on the relevance of dECM to OA. During the search process, multiple keyword combinations related to dEMC and OA were used to maximize the retrieval of relevant research. These keyword combinations included not only the core terms (e.g., “dEMC” and “OA”), but also related synonyms, variants, and potential research directions (e.g., “decellularized scaffold”, ‘joint degeneration’). The time frame of the search ranged from January 1, 2010 to November 1, 2024. After completing the searches, the two researchers independently compiled and compared the results of their respective searches. If there were discrepancies, the researchers reevaluated their search steps, strategies, and database settings to ensure the accuracy and consistency of the results. The detailed search formulas used by the researchers are shown below. The search formulas were customized for each database. At the same time, Our study strictly adhered to the PRISMA (Preferred Reporting Items for Systematic Reviews and Meta-Analyses) guidelines, which represent the gold standard for reporting systematic reviews.

Pubmed:

((decellularized extracellular matrix) OR (decellularisation) OR (decellularized scaffold) OR (decellularized biomaterial) OR (decellularized matrix)) AND ((osteoarthritis) OR (arthritis)).

Embase:

#1 ‘Decellularized matrix’ OR ‘decellularized scaffold’ OR ‘decellularized tissue’ OR ‘decellularized extracellular matrix’ OR ‘decellularized biomaterial’.

#2 ‘Osteoarthritis’ OR ‘degenerative joint disease’ OR ‘OA’ OR ‘arthrosis’ OR ‘joint degeneration’.

#3 #1 AND #2.

### Bibliometric analysis

To visually display the current publication status and trends of relevant research, VOSviewer (Version 1.6.19, Leiden University, The Netherlands) and Microsoft Office Excel 365 (Washington, DC, USA) were utilized for bibliometric analysis after duplicates removed. Herein, VOSviewer was used for keyword co-occurrence analysis. At visualization proceeding, the minimum number of co-occurrences for a keyword was set to 5, while normalization method was based on the association strength. All other options were set to default. Additionally, Microsoft Office Excel 365 was applied for quantitative analysis and visualization of the publication years of the articles.

### Inclusion and exclusion criteria

In this study, two researchers independently screened articles based on their titles, abstracts, and full texts. Any discrepancies between the two researchers were resolved through discussion to reach a consensus. If consensus could not be reached, a third senior researcher made the final decision following a group discussion.

#### Inclusion criteria

- Animal models of OA-related cartilage defects were involved to study the relationship between dECM and OA.
- Report Studies on the tissue repair function of dECM and its derivatives in cartilage defects associated with OA.
- The study contains a comparison of the treatment group with the control group.
- Contains complete data for the ICRS or OARSI scoring system to assess treatment effects and allow for effect size calculations.
- The type of study was a preclinical experimental animal study.
- Original research papers (excluding reviews, systematic evaluations, case reports, etc.)
- Studies that provide sufficient raw data for effect size calculations.
- Literature in English.

#### Exclusion criteria

- Studies not using animal models of OA-related cartilage defects or not investigating the dECM-OA relationship.
- Studies not reporting the tissue repair function of dECM/derivatives in OA-associated cartilage defects.
- Studies lacking comparison between treatment and control groups.
- Studies without complete ICRS or OARSI scoring data for treatment effect assessment and effect size calculations.
- Non-preclinical studies (e.g., clinical trials, in vitro studies) or studies not using animal models.
- Non-original research (reviews, systematic evaluations, case reports, etc.)
- Studies with insufficient raw data for effect size calculations.
- Non-English literature.

### The literature quality assessment

To assess the quality of the included studies, we applied the Cochrane Risk of Bias Tool and Systematic Review Centre for Laboratory Animal Experimentation (SYRCLE) Risk of Bias Tool. Specifically, studies with a high risk of bias (e.g., unclear randomization methods or inadequate blinding) and studies with a high attrition rate (over 20%) that did not properly handle missing data were excluded. Additionally, using the SYRCLE Risk of Bias Tool, studies that did not perform random allocation or blinding, and did not clearly describe these processes, were excluded. Studies with a “high” risk of bias or those with uncorrectable bias factors were also excluded from the analysis if these issues could not be addressed through other means. In addition to this, the methodological quality of the included studies was assessed using the Downs and Black Checklist, a validated tool for assessing randomized and non-randomized studies in five domains: reporting (10 items), external validity (3 items), internal validity - bias (7 items), internal validity - confounding (6 items) and statistical power (1 item). With the exception of the statistical power item (0–5 points), each item was scored as 0 (no/not clear) or 1 (yes), with a total score ranging from 0 to 32, with higher scores indicating better quality. Two independent reviewers conducted the assessment, resolving disagreements through discussion or third-party consultation if necessary. Studies were then categorized as high quality (≥ 20 points), moderate quality (15–19 points), and low quality (< 15 points) according to established thresholds to facilitate comparisons across studies.

### Data extraction and processing

To evaluate the tissue repair functions of dECM and relative derivatives in cartilage defects associated with OA, the ICRS and OARSI scores were respectively extracted from the literatures. Specific to literature without available original data, the webplotdigitizer software (version: 4.2) was employed to determine data based on figures.

### Statistical analysis and bias detection

In this meta-analysis, we used the Weighted Mean Difference (WMD) as the effect size to combine the results of continuous data from individual studies. WMD is suitable for studies with similar measurement scales and allows for the calculation of the mean differences between groups while considering sample size weighting. Statistical analysis was performed using Stata software (version 18.0). To assess heterogeneity, we first measured the *I*² statistic to evaluate the degree of variation between studies. If *I*²was less than 50% and the *P*-value was greater than 0.1, indicating low heterogeneity, a fixed effects model was applied, assuming a common true effect size across studies. If *I*²was greater than 50% and the *P*-value was less than 0.1, indicating significant heterogeneity, a random effects model was used, which accounts for the variations between studies. To ensure the robustness of the findings, a sensitivity analysis was conducted by leave-one-out method to evaluate their impact on the overall effect size. Large changes in effect size after exclusion suggested that certain studies had a substantial influence on the results. Additionally, due to the observed heterogeneity, a subgroup analysis was performed to explore the effects of different study characteristics, such as study design, sample size, and intervention methods, on the overall effect size. This allowed us to identify potential factors that might influence the results and provide more precise conclusions.

## Results

### Inclusion of literature and general information on the study

**Fig. 1:**
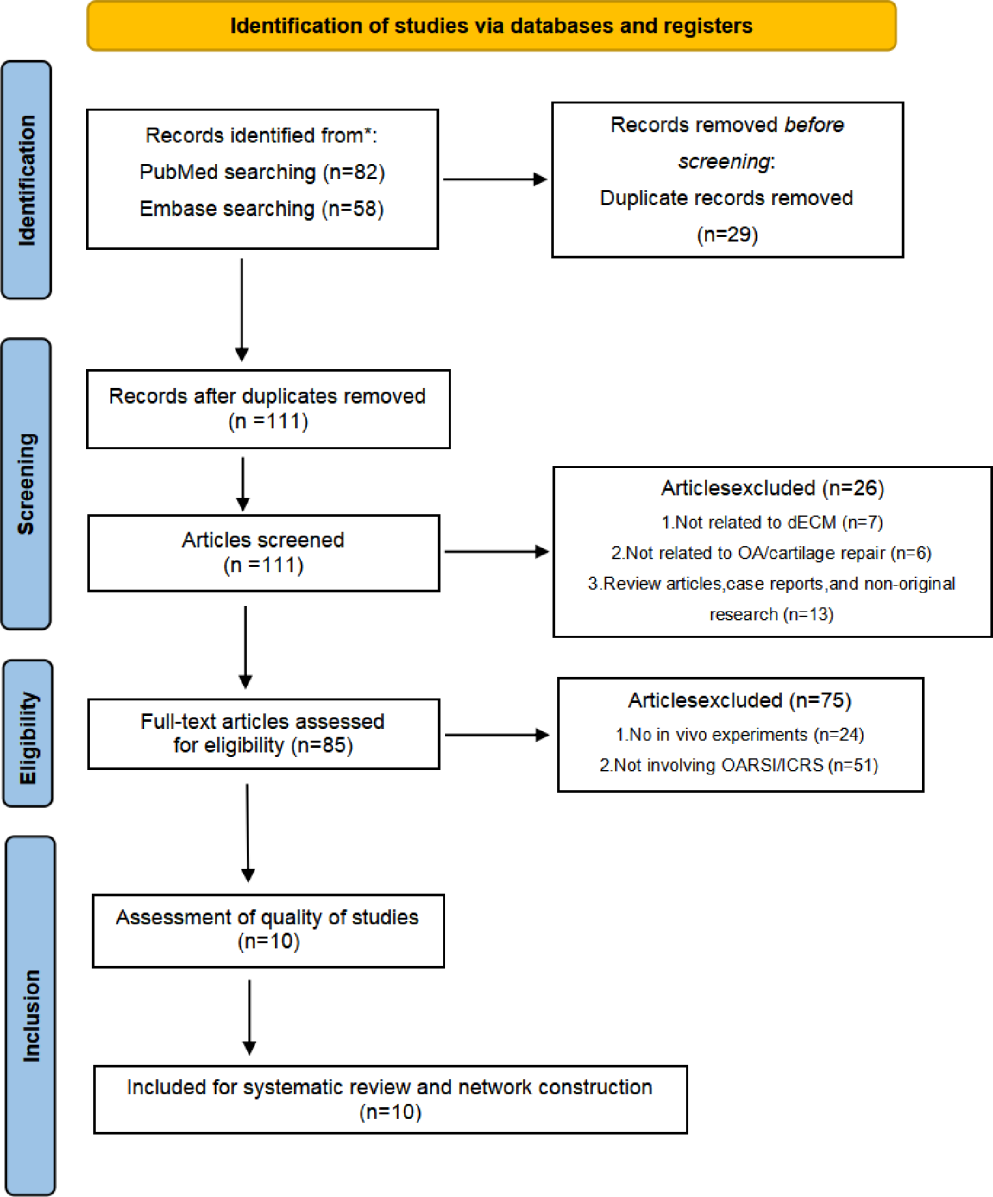
PRISMA flow diagram. *Consider, if feasible to do so, reporting the number of records identified from each database or register searched (rather than the total number across all databases/registers). **If automation tools were used, indicate how many records were excluded by a human and how many were excluded by automation tools. Source: Page MJ, et al. BMJ 2021;372:n71. doi: 10.1136/bmj.n71. This work is licensed under CC BY 4.0. To view a copy of this license, visit https://creativecommons.org/licenses/by/4.0/

As shown in Fig. 1, based on the preliminary searches in PubMed and Embase, a total of 82 and 58 articles were identified. After deduplication and screening, 10 studies were finally included [28–37] to further investigate the therapeutic effects of dECM in OA (Table 1). Meanwhile, the dECM preparation methods of each study are summarized in Table 2.

**Table 1:** Characteristics of studies included in the meta-analysis

Author	Year	Country	duration of model induction	Animal species	gender of animals	animal body weight	Assessment index	Sample size		Intervention	
								E	C	E	C
Chen et al.	2021	China	4w and 8w	Rats	Male	260 ± 10 g	OARSI	6	6	100 ml of 40% [w/v] dECM-CS compound	100 mlof chitosan
Choi et al.	2023	South Korea	8w and 12w	Rats	Male	240–290 g	ICRS	8	8	isolated dECMs	DPBS
Zhang et al.	2024	China	6w and 12w	Rabbits	NA	3.0–3.5 kg	ICRS	10	10	DMS-CBD + N-SDF1a	N-SDF1a
Wu et al.	2025	China	6w	Rats	Male	280–300 g	OARSI	10	10	50µL of dECMs	50µL of saline solution
Zhang et al.	2019	China	6w and 12w	Rabbits	Female	NA	ICRS	8	8	ECM	PBS
Zhang et al.	2018	China	24w and 36w	Goats	Male	30 ± 5 kg	ICRS	8	8	Celle-scaffold	Microfracture(nontreated)
Park et al.	2016	South Korea	2w and 4w	Rabbits	NA	NA	ICRS	4	4	CECM membranes + PTCD	PTCD
Meng et al.	2024	China	4w and 8w	Rats	NA	220–260 g	ICRS	4	4	dECM	gelatin and PBS
Gelse et al.	2017	Germany	6w and 26w	Sheep	Female	70–80 kg	OARSI	4	4	lateral meniscectomy (LMX) and lateral meniscal allografts (LMA)	nontreated
Zhu et al.	2020	China	4w and 8w	Rats	NA	220–260 g	ICRS	4	4	GelMA/dECM composite hydrogel	PBS

**Table 2:** Characteristics of dECM Preparation methods across included studies

Author	Year	Country	Tissue source	Decellularization method	ECM retention	Residual DNA content
Chen et al.	2021	China	Rat knee cartilage	Combination of physical, chemical, and enzymatic methods	GAGs (*P* > 0.05)	DNA content significantly reduced
Choi et al.	2023	South Korea	Human induced pluripotent stem cell-derived chondrocyte ECM	Combination of chemical, and enzymatic methods	Retains most GAGs and collagen	DNA content significantly reduced
Zhang et al.	2024	China	Rabbit meniscus	Chemical process	Retains most GAGs and collagen	DNA content significantly reduced
Wu et al.	2025	China	SD rat femoral cartilage	Combination of physical, chemical, and enzymatic methods	Retains most GAGs and collagen	DNA content significantly reduced
Zhang et al.	2019	China	New Zealand White rabbit femoral trochlear groove	Combination of chemical, and enzymatic methods	82.4% GAGs, 82.8% collagen retained	10%
Zhang et al.	2018	China	Caprine femoral condyle cartilage	Physical process	Preserves abundant cartilage extracellular matrix components and signaling molecules	DNA content significantly reduced
Park et al.	2016	South Korea	Porcine cartilage tissue	Combination of chemical, and enzymatic methods	Preserves natural articular cartilage components	NA
Meng et al.	2024	China	SD rat femoral cartilage	Combination of physical, chemical, and enzymatic methods	Retains most GAGs and collagen	DNA content significantly reduced
Gelse et al.	2017	Germany	Sheep meniscus	Chemical process	Retains the collagen network but removes proteoglycans	NA
Zhu et al.	2020	China	Porcine cartilage tissue	Combination of physical, chemical, and enzymatic methods	Preserves the natural microenvironment of the cartilage ECM to support chondrogenic induction of stem cells	DNA content significantly reduced

OARSI and ICRS scores were primarily obtained from available supplemental files. For studies that did not provide original data, data extraction was conducted using webplotdigitizer software based on the images in the text, enabling further analysis.

### Bibliometric analysis

**Fig. 2:**
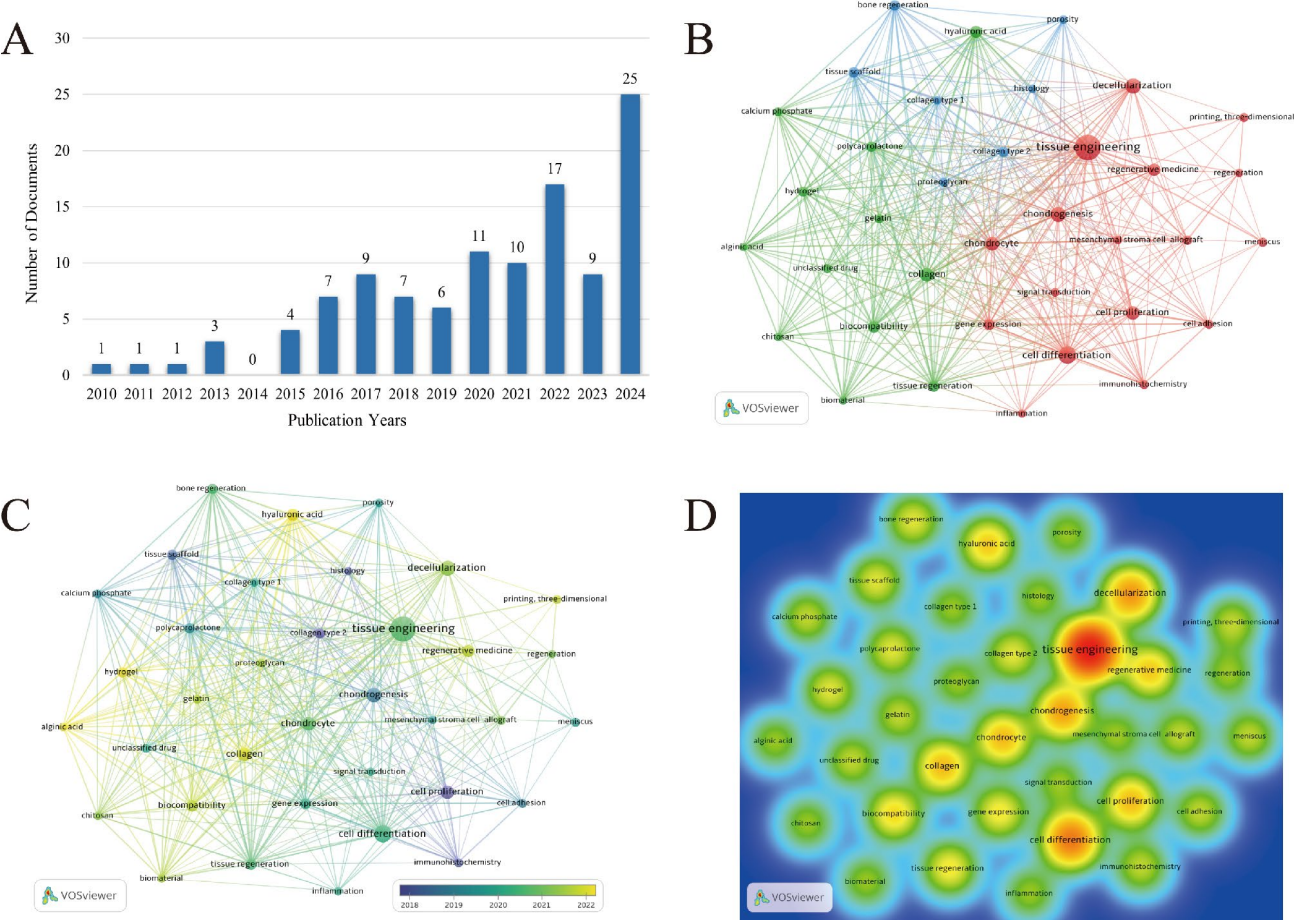
Bibliometric Analysis. (**A**) Distribution of publications by year from 2010 to 2024. (**B**) Network visualization mapping of the keywords. The size of the node is proportional to the frequency of the keyword. The colors of nodes and lines represent different keyword clusters, and the thickness of the connecting line indicates the strength of the co-occurrence link of the keyword. (**C**) Overlay visualization mapping of keywords. The color of each node corresponds to the average publication year of the keyword. The size of a note is proportional to the frequency of the keyword. (**D**) Density visualization mapping of the keywords. The redder the node, the higher the frequency of the corresponding keyword co-occurrence

According to the comprehensive screening, there were 111 papers published from 2010 to 2024. As shown in Fig. 2A, the number of papers exhibited an overall upward trend during this period, with a more significant increase after 2020, reaching a peak of 25 papers in 2024.

In the co-occurrence analysis, keywords were defined as words that appeared more than five times in the titles or abstracts of all papers and were selected and analyzed using VOSviewer. As illustrated in Fig. 2B, the 36 identified keywords were primarily categorized into three clusters: cluster 1 (red): tissue engineering; cluster 2 (blue): scaffold materials; and cluster 3 (green): biomaterials. These results highlight the most prominent research topics related to dECM and OA to date. In the “tissue engineering” cluster, the main keywords included tissue engineering, regenerative medicine, and regeneration. For the “scaffold materials” cluster, the commonly used keywords were tissue scaffold and porosity. In the “biomaterials” cluster, the main keywords used were collagen, polycaprolactone, and biocompatibility. These results indicate that the most prominent areas related to dECM and osteoarthritis research include the above three directions.

According to Fig. 2C, VOSviewer colored all keywords based on their average frequency of appearance in the published papers. Specifically, blue indicates that the keywords appeared relatively early, while yellow indicates more recent emergence. As shown in Fig. 2C, the research trends of most studies in the three clusters changed from cell proliferation and chondrogenesis to cell differentiation, tissue engineering, and collagen. This suggests that future research hotspots might lie in the areas of cell differentiation and biomaterials.

Figure 2D reflects the research hotspots and trends in the field of tissue engineering, indicating that the field is developing towards interdisciplinary integration. The research hotspots mainly focus on four aspects: tissue engineering, topics related to cell and molecular biology, topics related to materials science, and biomaterials and scaffolds. The research trends in recent years have primarily concentrated on the following three aspects: regenerative medicine and tissue regeneration, 3D printing technology, and immune response and biocompatibility.

### Quality assessment of literature

The methodological quality of the 10 included studies was evaluated using three complementary tools: the Cochrane Risk of Bias Tool for randomized controlled trials, the SYRCLE Risk of Bias Tool for animal studies, and the Downs and Black checklist for broader methodological assessment. Detailed risk of bias evaluations are presented in Table 3, while the comprehensive Downs and Black quality scores are shown in Table 4.

**Table 3:** Assessment of quality of studies

Study(years)	Selection bias			Performance bias		Detection bias	Attrition bias	Reporting bias	other
	Sequence generation	Baseline characteristics	Allocation concealment	Random housing	Blinding	Random outcome assessment	Incomplete outcome data	Selective outcome reporting	Other sources of bias
Chen et al. (2021)	Unclear risk	Unclear risk	Unclear risk	low risk	Unclear risk	low risk	low risk	low risk	Unclear risk
Choi et al. (2023)	Unclear risk	Unclear risk	Unclear risk	Unclear risk	Unclear risk	low risk	low risk	low risk	Unclear risk
Zhang et al. (2024)	Unclear risk	low risk	Unclear risk	low risk	Unclear risk	low risk	low risk	low risk	Unclear risk
Wu et al. (2025)	Unclear risk	Unclear risk	Unclear risk	Unclear risk	Unclear risk	low risk	low risk	low risk	Unclear risk
Zhang et al. (2019)	Unclear risk	low risk	Unclear risk	low risk	Unclear risk	low risk	low risk	low risk	Unclear risk
Zhang et al. (2018)	low risk	low risk	Unclear risk	low risk	low risk	Unclear risk	low risk	low risk	Unclear risk
Park et al. (2016)	Unclear risk	low risk	Unclear risk	low risk	low risk	Unclear risk	low risk	low risk	Unclear risk
Meng et al. (2024)	Unclear risk	low risk	Unclear risk	low risk	Unclear risk	low risk	low risk	low risk	Unclear risk
Gelse et al. (2017)	low risk	low risk	Unclear risk	low risk	Unclear risk	low risk	low risk	low risk	Unclear risk
Zhu et al. (2020)	Unclear risk	low risk	Unclear risk	low risk	Unclear risk	low risk	low risk	low risk	Unclear risk

**Table 4:** Downs and black checklist

Authors and year of publication	Reporting											External validity			Internal validity - bias						Internal validity	Internal validity - confounding						POWER	Total	Quality
	1	2	3	4	5	6	7	8	9	10	11	12	13	14	15	16	17	18	19	20	21	22	23	24	25	26	27	28		
Chen et al. (2021)	1	1	0	1	1	1	0	1	1	1	1	0	1	1	1	0	1	1	1	0	1	1	1	1	1	0	1	3	24	high
Choi et al. (2023)	1	1	1	1	0	1	1	1	1	1	0	1	1	1	0	1	1	1	1	0	1	1	0	1	1	1	1	2	24	high
Zhang et al. (2024)	1	0	1	1	1	1	1	0	1	1	1	1	0	1	1	1	1	0	1	1	1	1	0	1	1	1	1	4	26	high
Wu et al. (2025)	1	1	1	0	1	1	1	1	0	1	1	1	1	0	1	1	1	1	0	1	1	1	1	0	1	1	1	5	27	high
Zhang et al. (2019)	1	0	1	0	1	1	0	1	0	1	1	0	1	0	1	0	1	1	0	1	0	1	1	0	1	0	1	3	19	medium
Zhang et al. (2018)	0	1	1	0	1	0	1	1	0	1	0	1	1	0	1	0	1	1	0	1	0	1	1	0	1	0	1	4	20	high
Park et al. (2016)	1	1	1	1	1	0	1	1	1	1	0	1	1	1	1	0	1	1	1	1	0	1	1	1	1	0	1	3	25	high
Meng et al. (2024)	1	1	0	1	0	1	0	1	1	0	1	0	1	1	0	1	0	1	0	1	1	0	1	0	1	1	0	2	18	medium
Gelse et al. (2017)	1	1	0	1	1	1	1	0	1	1	1	1	0	1	1	1	1	0	1	1	1	1	0	1	1	1	1	2	24	high
Zhu et al. (2020)	1	0	1	1	0	1	0	1	0	1	1	0	1	0	1	1	0	1	0	1	0	1	1	0	1	0	1	1	17	medium

### Meta-analysis reveals significant therapeutic effects of dECM in OA

**Fig. 3:**
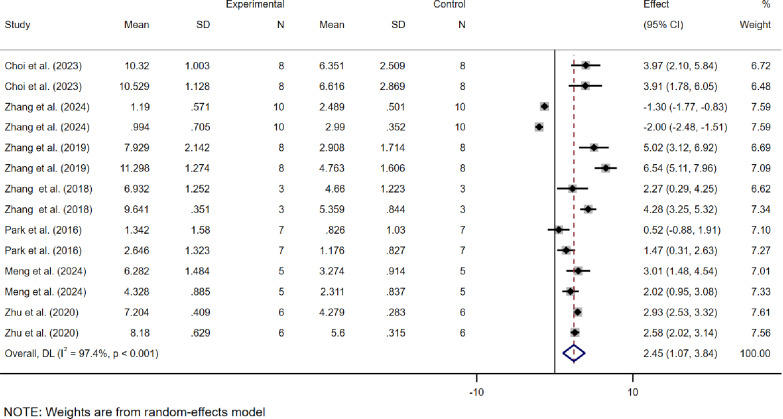
Meta-analysis forest plots based on ICRS scores

The WMD was used as the effect size because the scoring systems across studies were consistent, allowing direct comparison of means between groups. Among the 7 studies based on ICRS scores, as most studies included data from multiple groups with different modeling times and varying lesion sites, multiple independent sets of data verifying the efficacy of dECM were extracted from individual experiments. A total of 14 ICRS-related datasets were obtained, showing an WMD of 2.45 (95% CI: 1.07 to 3.84) between the dECM treatment group and the control group, with high heterogeneity (*I*² = 97.4%) (Fig. 3).

**Fig. 4:**
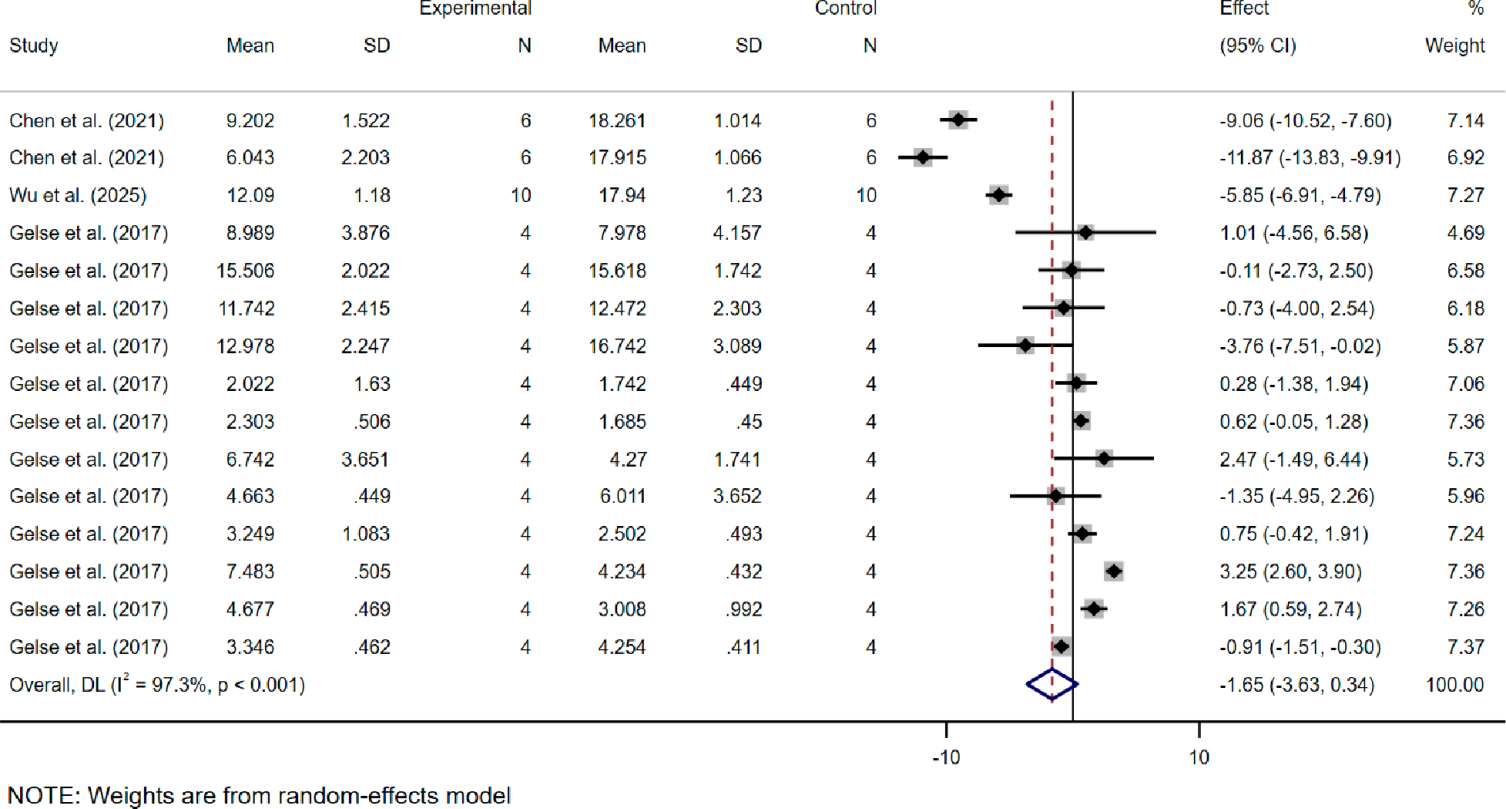
Meta-analysis forest plots based on OARSI scores

Regarding the OARSI score (Fig. 4), the study includes multiple datasets derived from different modeling times and various levels of analysis, allowing the extraction of independent datasets from each experiment to evaluate the efficacy of dECM. A total of 15 research datasets were collected, with results indicating a WMD of -1.65 (95% CI: -3.63 to 0.34), accompanied by high heterogeneity (*I*² = 97.3%).

Therefore, to clarify the source of heterogeneity, we conducted a subgroup analysis. By grouping based on different experimental species, we further investigated the chondroprotective effects of dECM and its tissue repair function under varying conditions.

### Subgroup analysis based on different experimental animal species

**Fig. 5:**
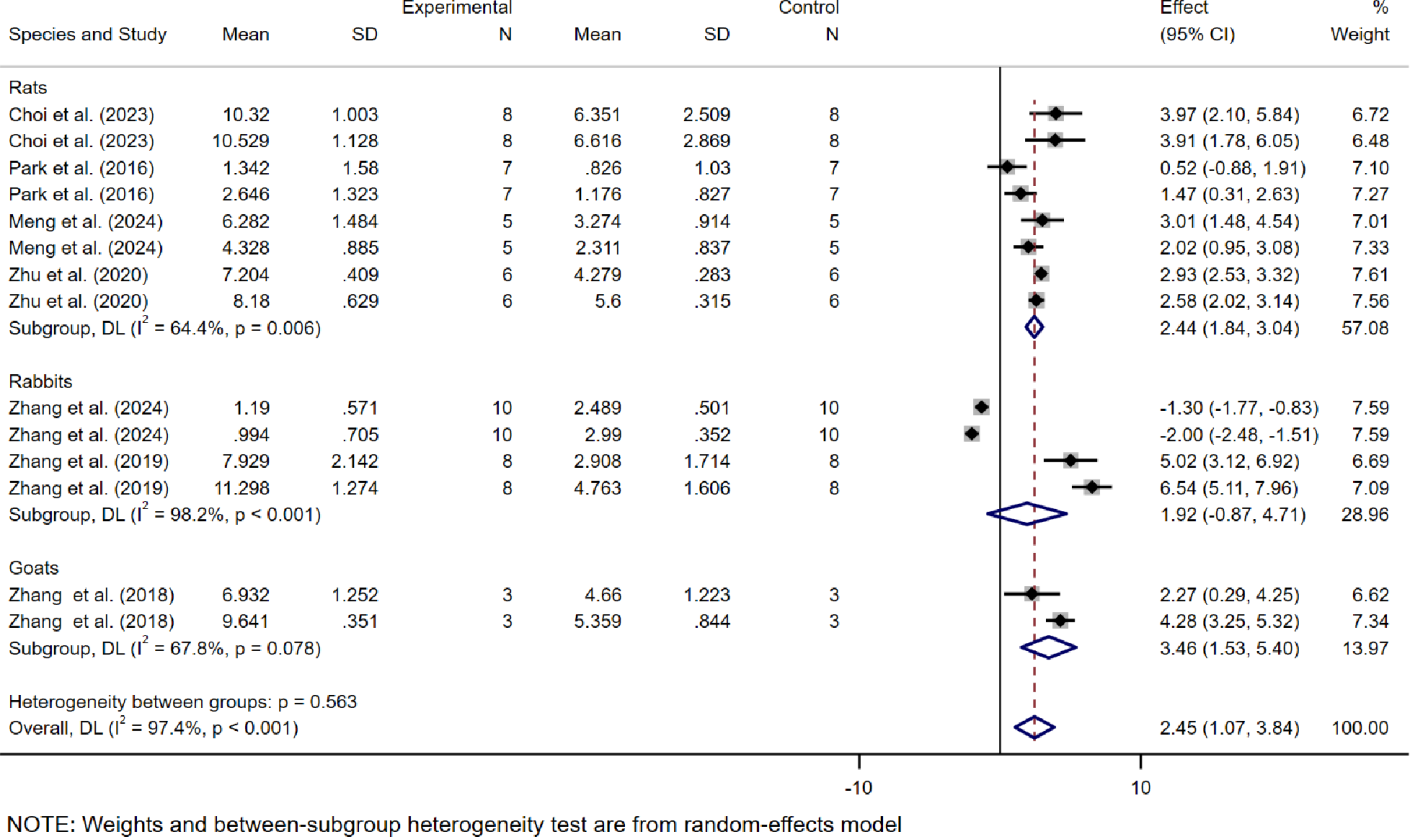
The subgroup analysis based on ICRS scores according to experimental animal species

Subgroup analysis indicated that, based on ICRS studies, dECM demonstrated significant efficacy in rats (WMD = 2.44, 95% CI: 1.84–3.04, *I*² = 64.4%) and goats (WMD = 3.46, 95% CI: 1.53–5.40, *I*² = 67.8%), with moderate heterogeneity in both groups. However, its efficacy was limited in the rabbit subgroup (WMD = 1.92, 95% CI: -0.87-4.71, *I*² = 98.2%) and showed high heterogeneity (Fig. 5).

**Fig. 6:**
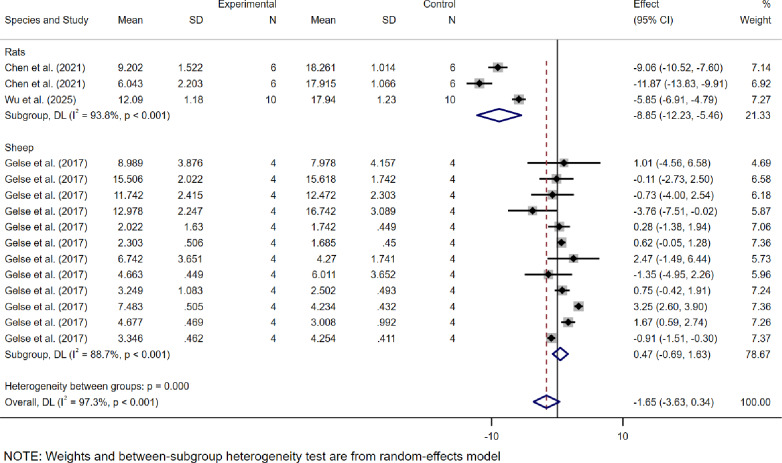
The subgroup analysis based on OARSI scores according to experimental animal species

Based on the OARSI study cohort, the rat subgroup showed significant therapeutic effects of dECM (WMD = -8.85, 95% CI: -12.23 to -5.46, *I*²= 93.8%), whereas no significant effect was observed in the sheep subgroup (WMD = 0.47, 95% CI: -0.69 to 1.63, *I*²= 88.7%). Both subgroups exhibited high heterogeneity (Fig. 6).

### Sensitivity analysis

**Fig. 7:**
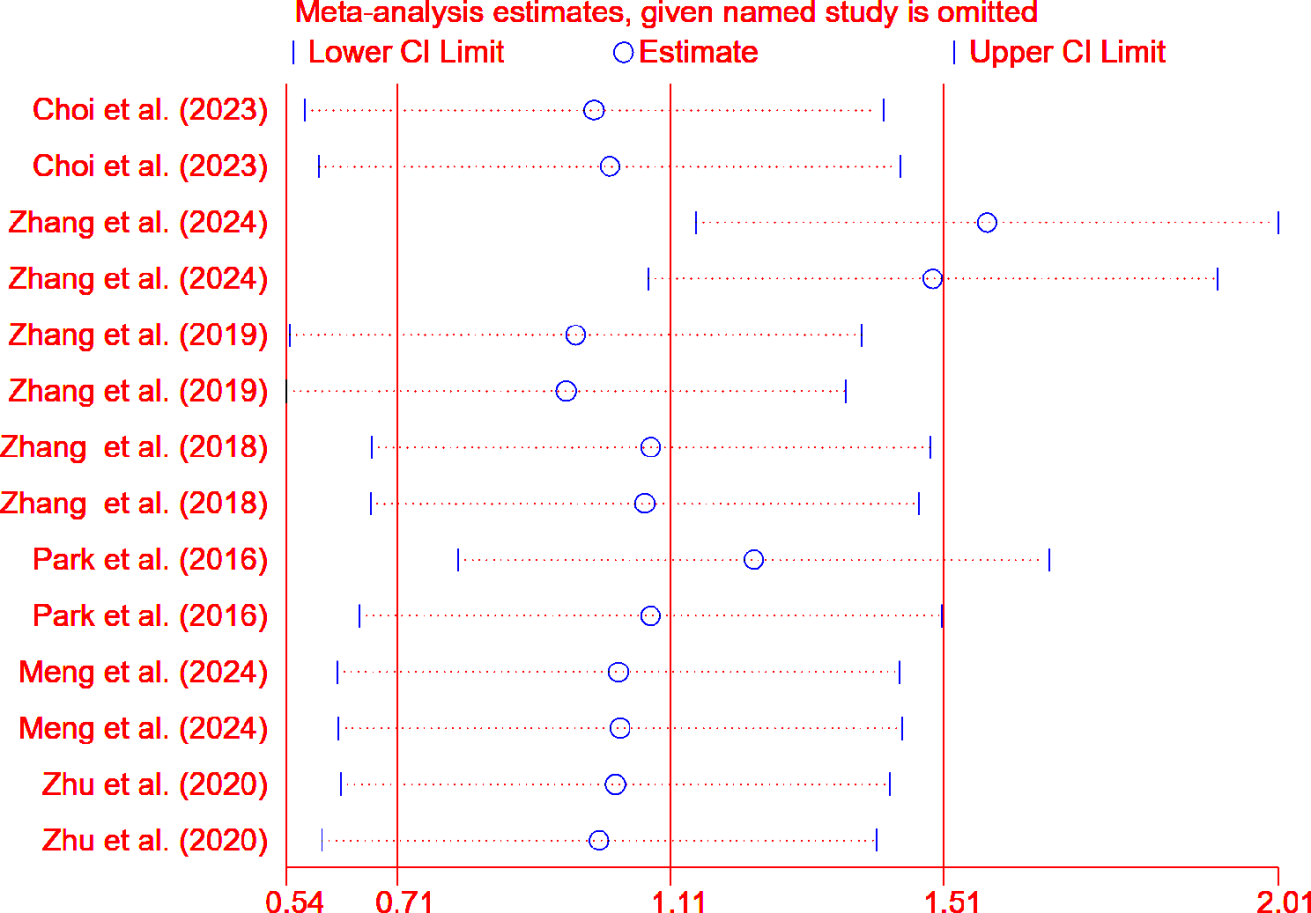
Sensitivity analysis results based on ICRS scores

**Fig. 8:**
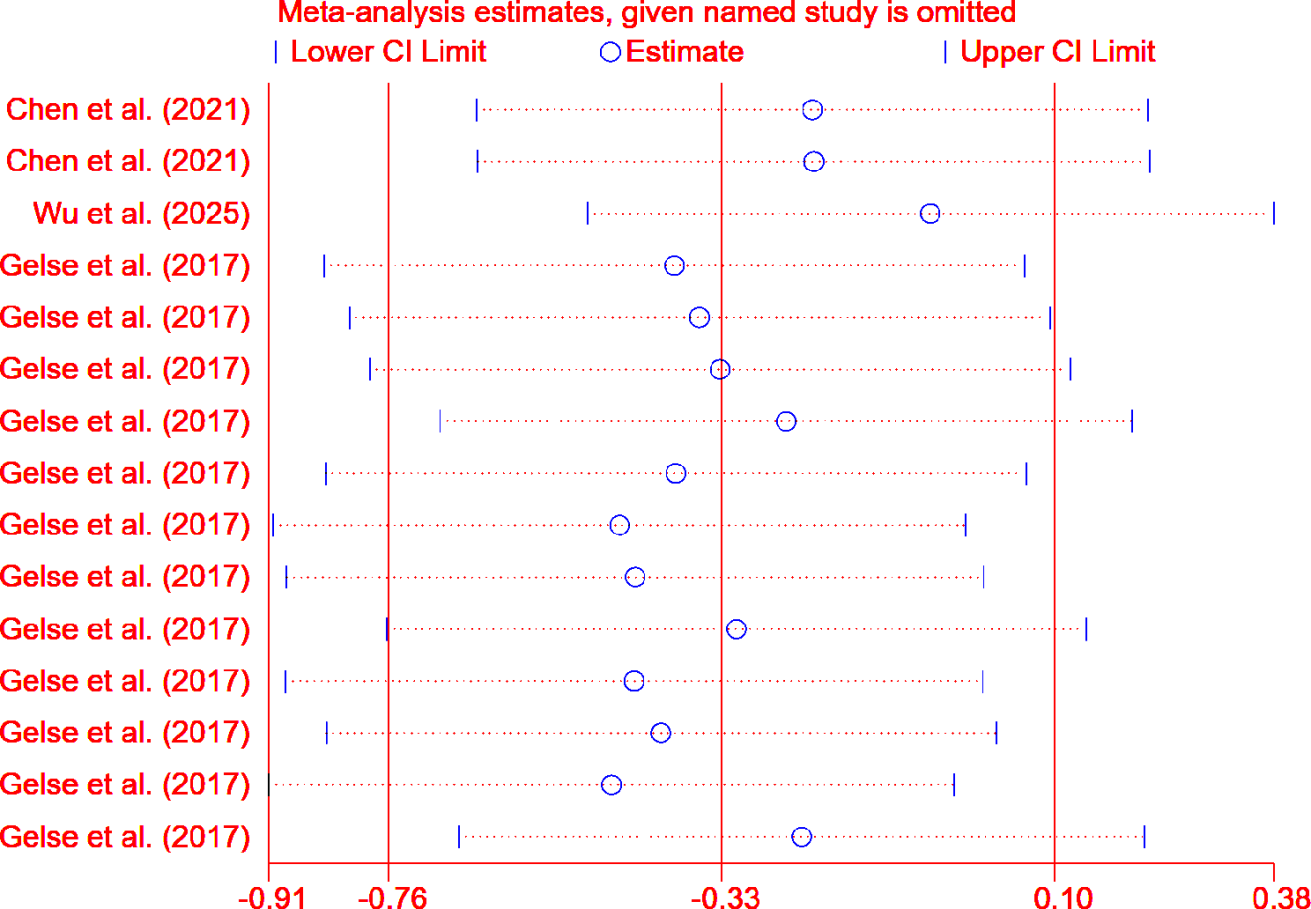
Sensitivity analysis results based on OARSI scores

For the ICRS scoring system (Fig. 7), excluding the data from Zhang et al. (2024) significantly affects the overall effect size (WMD = 1.578, 95% CI: 1.149 to 2.007) and (WMD = 1.497, 95% CI: 1.078 to 1.917). However, for the OARSI scoring system (Fig. 8), excluding any single study does not significantly affect the overall effect size.

### Bias analysis

**Fig. 9:**
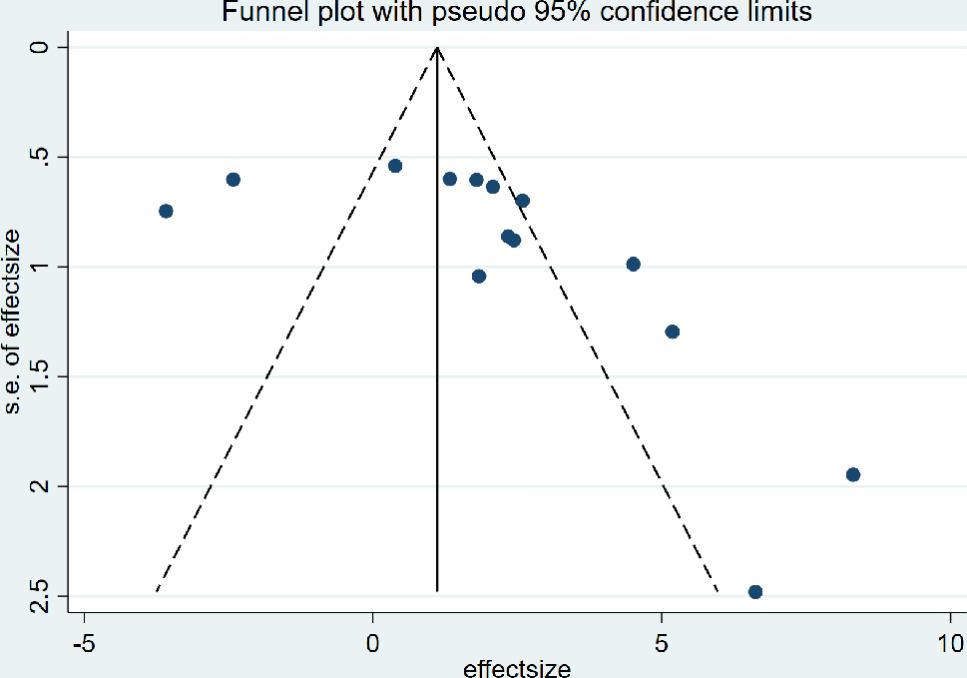
Funnel plots based on ICRS scores

**Fig. 10:**
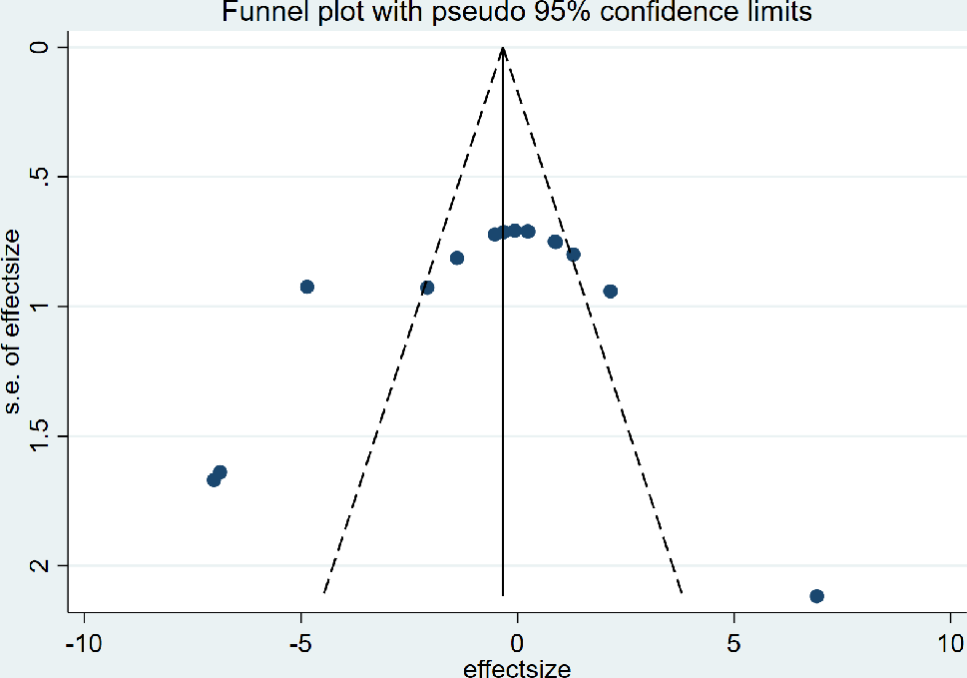
Funnel plots based on OARSI scores

The funnel plots based on ICRS/OARSI studies displayed a symmetrical distribution of studies around the center line. Despite slight asymmetry suggesting potential minor biases or omissions of small sample studies, the overall results indicate that the therapeutic effect of dECM on OA is not significantly influenced by publication bias. The results of the bias analysis confirm the reliability of the study conclusions (Figs. 9 and 10).

## Discussion

This study focuses on exploring novel therapeutic strategies to address the limitations of current OA treatments, particularly in promoting cartilage regeneration and slowing disease progression. In recent years, dECM materials have attracted widespread attention due to their unique advantages in tissue repair and regeneration. With the continuous in-depth research on dECM materials, the number of related publications has been steadily increasing year by year, and the scope and depth of research continue to expand. According to statistics from the past decade, as many as 105 papers on dECM materials have been published, covering core areas such as tissue engineering, regenerative medicine, and decellularization. Moreover, the study of cellular mechanisms, particularly processes like chondrogenesis, cell differentiation, and cell proliferation, has become a focal point, as these mechanisms are crucial for the effectiveness of dECM materials in tissue regeneration [23]. Previous studies have demonstrated that decellularized extracellular matrix (dECM) possesses unique biochemical properties that can support cellular activities in cartilage repair, offering a novel therapeutic potential for osteoarthritis (OA) treatment. Our systematic review and meta-analysis synthesized data from various animal studies to evaluate the efficacy of dECM in enhancing cartilage regeneration and mitigating inflammation in OA. By comparing treatment outcomes (measured using standardized scoring systems) with control groups, we aimed to provide a clearer understanding of the therapeutic effects of dECM. The findings of this study may pave the way for harnessing the regenerative capacity of dECM in OA therapy. Future research should focus on optimizing dECM-based scaffolds to improve their mechanical properties and biocompatibility, ensuring their ability to withstand repetitive joint stresses. Additionally, exploring the combination of dECM with growth factors or stem cells may further enhance cartilage regeneration while reducing the risk of fibrocartilage formation. Moreover, long-term clinical trials are necessary to assess the durability and safety of dECM-based therapies in human patients. The clinical significance of dECM in cartilage repair is substantial. By providing a biomimetic microenvironment that supports chondrocyte function and stem cell differentiation, dECM has the potential to restore the structural integrity and functionality of articular cartilage. This approach may offer a more effective alternative to current treatments, such as nonsteroidal anti-inflammatory drugs (NSAIDs) and joint replacement surgery, which often provide only temporary relief or are associated with significant adverse effects. Combining dECM with other therapeutic strategies, such as intra-articular injections of hyaluronic acid or growth factors, could further enhance the overall efficacy of OA treatment. Ultimately, dECM-based therapies hold promise for improving the quality of life in OA patients by delivering a more sustainable and regenerative solution [17, 22, 23].

The ICRS and OARSI are two canonical scoring systems in evaluating OA therapeutic effect. Specifically, the ICRS scoring system primarily evaluates cartilage repair quality through arthroscopy or direct observation, making it suitable for rapid quantitative analysis of repair tissue surface smoothness, hardness, and integration in experimental animals. It is widely used in animal cartilage repair research and can intuitively reflect the effects of different treatment methods [38]. The higher the ICRS score, the better the cartilage repair outcome. On the other hand, the OARSI score provides an in-depth analysis of OA lesions through histological sections, assessing features such as cartilage degeneration, osteophyte formation, and subchondral bone plate changes. It is especially suitable for studying early lesion characteristics and evaluating the effects of therapeutic interventions [39]. The OARSI score focuses on evaluating the deeper pathological features of lesions, with higher scores indicating more severe damage.

First, in this study, only 10 studies were finally included in the meta-analysis by systematic search of PubMed (82 articles) and Embase (58 articles), and the main reasons for the inclusion of only 10 studies included [1] strict adherence to the pre-set inclusion criteria, in particular, the requirement that studies must include both animal experimental data and the results of the ICRS/OARSI scores, and thus excluding studies that did not evaluate efficacy by the ICRS/ OARSI score to evaluate efficacy (*n* = 51); [2] excluded in vitro studies (*n* = 24) lacked in vivo efficacy evaluation although they explored the biological mechanisms of dECM. Such rigorous screening ensured the reliability of the included studies and data analysis.

Next, this study combines the ICRS and OARSI scoring systems and evaluates the effects of dECM in the treatment of OA through a meta-analysis. The results show a significant difference between the dECM treatment group and the control group in the ICRS scores, with statistical significance (WMD = 2.45, 95% CI: 1.07 to 3.84, *I*² = 97.4%). This finding suggests that dECM significantly promotes cartilage repair in OA, particularly in improving the quality of the cartilage surface. However, although the OARSI scores also showed efficacy, it did not reach statistical significance. Specifically, the mean difference in OARSI scores between the dECM treatment group and the control group was − 1.65 (95% CI: -3.63 to 0.34, *I*² = 97.3%), which did not reach significance. The reason for this may be that the ICRS score primarily focuses on the macroscopic repair of cartilage, which is sensitive to changes in the surface quality of cartilage, while the OARSI score assesses more complex histological changes in OA, involving intricate pathological processes. Although dECM significantly promotes cartilage surface repair, its effects on slowing cartilage degeneration and the formation of osteophytes, among other deeper pathological changes, may be more limited. Therefore, the differences between ICRS and OARSI scores may stem from their differing focuses and sensitivities: ICRS is more focused on surface repair, while OARSI assesses more complex histological changes.

At the same time, the high heterogeneity in the meta-analysis (ICRS *I*² = 97.4%, OARSI *I*² = 97.3%) suggests that there may be differences between studies in experimental design, sample characteristics, or intervention protocols, which could affect the stability and consistency of the results. In particular, differences in experimental animal species, and the variations in intervention measures across studies could be major factors contributing to the heterogeneity. For example, the choice of experimental animals (such as mice, rabbits, or goats) could impact the effectiveness of dECM, as different species have distinct physiological and pathological characteristics. Furthermore, the variations in intervention measures used in the studies (such as the dosage of dECM or the method of administration) could also contribute to the heterogeneity of the results.To further investigate the source of heterogeneity, we conducted a subgroup analysis. This analysis helps to better understand the key variables that influence the effectiveness of dECM treatment, thereby providing clearer directions for future research.

Regarding subgroup analysis based on the ICRS score, dECM demonstrated more significant efficacy with moderate heterogeneity in mouse and goat models (*I*²= 64.4% and 67.8%, respectively), which may be attributed to the relatively standardized experimental conditions of these models. The simplified physiological mechanisms and high metabolic activity in mouse models highlighted the role of dECM in OA cartilage repair, while goat models, with their closer physiological resemblance to humans, provide greater clinical relevance in treatment response. In rabbit models, the efficacy of dECM was limited, with extremely high heterogeneity (*I*² = 98.2%), suggesting that rabbit models may exhibit different physiological responses during OA cartilage repair compared to mice and goats, leading to reduced or unstable therapeutic effects of dECM. Analysis based on the OARSI score further indicated that mouse models are more sensitive to dECM treatment, whereas sheep models did not exhibit statistically significant efficacy. This may reflect interspecies differences in cartilage repair mechanisms and treatment response rates. The larger body size and more complex OA cartilage repair processes in sheep may require longer treatment durations or more frequent interventions. Meanwhile, the high heterogeneity (*I*² = 93.8% and 97.3%) suggests the need to improve experimental design and increase sample sizes to more accurately evaluate treatment effects across different species. In conclusion, dECM demonstrated more stable effects in OA cartilage repair in mouse and goat models, whereas studies on rabbit and sheep models require further optimization to comprehensively assess its potential efficacy.

Sensitivity analysis also demonstrated the robustness of the meta-analysis results based on both scoring systems. Although excluding specific datasets (e.g., data from Zhang et al. (2024)) had some impact on the statistical significance of the ICRS scores, the overall conclusion remained consistent and reliable. When the data from Zhang et al. (2024) were excluded, the pooled effect size reached its maximum, indicating that the results of this study had an adverse impact on the overall effect size. In Zhang et al. (2024), the treatment group using decellularized meniscal scaffolds (DMS) showed significantly lower efficacy compared to the control group. This result may reflect the suboptimal efficacy of DMS in the study, potentially due to weak responses of the experimental animals to DMS, issues with DMS quality or dosage, or differences in other experimental conditions. It is also possible that DMS requires combination with other therapeutic approaches to enhance its efficacy. Therefore, certain specific factors in Zhang et al. (2024) may have influenced the effectiveness of DMS in this experiment, leading to differences in therapeutic outcomes compared to the control group.

In summary, integrating dECM into the treatment strategies for cartilage repair in OA represents a paradigm shift in regenerative medicine. Its ability to support chondrocyte function and its immunomodulatory properties position it as a promising candidate for future clinical applications. Ongoing research and clinical trials are essential to fully elucidate the therapeutic mechanisms of dECM and establish standardized application protocols in clinical settings, with the ultimate goal of promoting cartilage repair and regeneration [28].

The limitations of this study warrant careful consideration, as they may influence the interpretation and application of the findings. Notably, the absence of wet lab experiments limits the direct biological validation of the therapeutic effects of dECM in cartilage repair. Additionally, the small sample sizes in certain included studies could compromise the robustness of the results, particularly in assessing variabilities in treatment response. Meanwhile, although this study conducted a systematic search through two authoritative databases, PubMed and Embase, there are still some limitations: first, limited by the number of databases searched, relevant studies in other specialized databases (e.g., Web of Science, Scopus, etc.) may be missed; second, there are variations in the scope of inclusion and search algorithms of different databases. The search strategy of a single platform may not be able to fully capture all relevant literature. These factors may have a certain impact on the comprehensiveness of the study results, and future research can be further improved by expanding the search scope and adopting a more comprehensive search strategy. Also as a dECM-based preclinical study, our work provides a valuable initial exploration of potential therapeutic mechanisms. However, the study still has obvious limitations - the translational relevance of dECM scaffolds to human physiology is not yet clear due to the lack of clinical validation. Current findings from in vitro experiments and animal models may not fully recapitulate the complexity of human pathologies, which somewhat limits the generalizability of the findings. inherent differences between the dECM platform and the natural human tissue microenvironment further highlight the need for future clinical trials. The need to include human subjects in follow-up studies will be decisive to validate the efficacy and safety observed in this dECM preclinical study. Furthermore, the potential inter-batch variability of dECM may introduce inconsistencies in the treatment effects observed across different studies, necessitating standardized protocols in future research endeavors. Meanwhile, future studies should also further explore the differences between scoring systems, particularly their applicability in clinical practice. Although existing studies suggest that dECM has therapeutic benefits to some extent, further long-term safety evaluations and rigorous clinical validations are needed to confirm its potential for widespread clinical application. In a word, Based on a meta-analysis of ICRS and OARSI scores, dECM and its related derivatives have been shown to exhibit significant cartilage repair functions in OA, offering potential for further exploration in the field of tissue regeneration.

## Electronic supplementary material

Below is the link to the electronic supplementary material.

Supplementary Material 1

## Data Availability

No datasets were generated or analysed during the current study.

## Abbreviations

OA: Osteoarthritis
dECM: Decellularized Extracellular Matrix
WMD: Weighted Mean Difference
OARSI: Osteoarthritis Research Society International
ICRS: International Cartilage Repair Society
